# Using Molecular Spectroscopic Techniques (NIR and ATR-FT/MIR) Coupling with Various Chemometrics to Test Possibility to Reveal Chemical and Molecular Response of Cool-Season Adapted Wheat Grain to Ergot Alkaloids

**DOI:** 10.3390/toxins15020151

**Published:** 2023-02-13

**Authors:** Haitao Shi, Peiqiang Yu

**Affiliations:** 1 College of Agriculture and Bioresources, University of Saskatchewan, Saskatoon, SK S7N 5A8, Canada; 2 College of Animal and Veterinary Sciences, Southwest Minzu University, Chengdu 610041, China

**Keywords:** spectroscopic techniques, chemometric, spectral pretreatments, the partial least squares regression (PLS), wavelength selection, mycotoxin quantification, cool-season adapted wheat

## Abstract

The objectives of this study were to explore the possibility of using near infrared (NIR) and Fourier transform mid-infrared spectroscopy—attenuated total reflectance (ATR-FT/MIR) molecular spectroscopic techniques as non-invasive and rapid methods for the quantification of six major ergot alkaloids (EAs) in cool-season wheat. In total, 107 wheat grain samples were collected, and the concentration of six major EAs was analyzed using the liquid chromatography-tandem mass spectrometry technique. The mean content of the total EAs—ergotamine, ergosine, ergometrine, ergocryptine, ergocristine, and ergocornine—was 1099.3, 337.5, 56.9, 150.6, 142.1, 743.3, and 97.45 μg/kg, respectively. The NIR spectra were taken from 680 to 2500 nm, and the MIR spectra were recorded from 4000–700 cm^−1^. The spectral data were transformed by various preprocessing techniques (which included: FD: first derivative; SNV: standard normal variate; FD-SNV: first derivative + SNV; MSC: multiplicative scattering correction; SNV-Detrending: SNV + detrending; SD-SNV: second derivative + SNV; SNV-SD: SNV + first derivative); and sensitive wavelengths were selected. The partial least squares (PLS) regression models were developed for EA validation statistics. Results showed that the constructed models obtained weak calibration and cross-validation parameters, and none of the models was able to accurately predict external samples. The relatively low levels of EAs in the contaminated wheat samples might be lower than the detection limits of the NIR and ATR-FT/MIR spectroscopies. More research is needed to determine the limitations of the ATR-FT/MIR and NIR techniques for quantifying EAs in various sample matrices and to develop acceptable models.

## 1. Introduction

Ergot alkaloids (EAs) are toxic compounds produced by *Claviceps* fungi species, which can parasitize the seed heads of some small grains and grasses during the time of flowering [1,2]. Th Numerous monocotyledonous plants can be attacked by those fungi, including durum wheat, oats, barley, rye, corn, forage grasses, etc. [3]. During the infection, the healthy grain or seed will be replaced by ergots (i.e., sclerotia). The ergots are brown to purple-black in color and usually contain high concentrations of EAs [4].

More than 40 different EAs have been reported. Generally, they could be classified into three groups, including clavinet alkaloids, peptide alkaloids, and lysergic acid derivatives [5]. Ergometrine, ergotamine, ergosine, ergocristine, ergocryptine, and ergocornine are the main EAs produced by *Claviceps* species [4]. Ergots are usually harvested from uncontaminated grass or grains. Even when the sclerotia were removed from wheat and rye samples by hand cleaning, EAs could still be found [6].

A widespread presence of ergot and EAs contamination in western Canadian cereals has been reported. In 1999, 4% of Western Durum and 12% of Canadian Western Red Spring wheat samples were positive for ergot, and similar outbreaks have been reported in Manitoba in 2005 and in all three Prairie provinces (Alberta, Saskatchewan, and Manitoba) in both 2008 and 2011 [7]. EAs can pose great health risks to humans and animals. For instance, EAs can harm the health and productivity of animals, such as lactation performance, growth, reproductive performance, pregnancy rates, sperm motility, etc. [8].

Due to analytical limitations, the monitoring of ergot contamination of grain is mainly focused on controlling the content of ergot bodies, while regulations for the concentration of individual EAs in grain are still unavailable [6]. However, the content of EAs and the proportion of individual EAs are extremely variable within ergot bodies and could be significantly affected by geographic regions, harvest time, crop species, and variety [8]. Up to date, the popular methods for the determination of EAs in agricultural commodities are based on wet chemistry, such as high-performance liquid chromatography, liquid chromatography-tandem mass spectrometry (LC-MS/MS), thin-layer chromatography, etc. [9,10]. These methods usually need professional technicians and are expensive and time-consuming.

Infrared (IR) spectroscopy has been widely used as a fast and noninvasive approach in feed and food research [10]. Moreover, the IR spectroscopy technique has been reported as a promising technique for the estimation of mycotoxins in agricultural commodities [11]. The commonly used infrared spectral data methods for transformation with various preprocessing techniques include: FD: first derivative; SNV: standard normal variate; FD-SNV: first derivative + SNV; MSC: multiplicative scattering correction; SNV-Detrending: SNV + detrending; SD-SNV: second derivative + SNV; SNV-SD: SNV + first derivative. Shi et al. (2019) [10] reported vibrational spectroscopy limitations for the barley study and suggested additional research on vibrational spectroscopy in grain research. Some studies have been conducted to explore the potential of infrared-based techniques in the ergot detection area. Roberts et al. (1997) [12] applied the NIR method for the quantitative analysis of ergovaline concentration in tall fescue. In another study, Vermeulen et al. (2012) [4] established a model for quantifying the content of ergot bodies (0–10,000 mg/kg) based on hyperspectral imaging techniques. In our previous study, Shi et al. (2019) [10] reported a barley study with vibrational spectroscopy. Nevertheless, the possibility of using a spectroscopic method for the fast prediction of major EAs in cool-season-adapted wheat with low heat units has not been explored.

The aim of this research was to explore the possibility of using near- and mid-infrared spectroscopy combined with different spectral pretreatments and spectral regions to quantify the six major EAs in western Canadian wheat grown under low heat unit (cold) climate conditions.

## 2. Results and Discussion

### 2.1. Statistic Values of EAs

According to the LC-MS/MS analysis, EAs were detected in 75 of the collected samples. Table 1 shows the statistical summary of the ergot alkaloids, including the concentration ranges, averages, standard errors, skewness, and variance. The mean concentrations for total EAs, ergotamine, ergosine, ergometrine, ergocryptine, ergocristine, and ergocornine were 1099.3, 337.5, 56.9, 150.6, 142.1, 743.3, and 97.5 μg/kg, respectively. Most of the positive samples contained relatively low levels of EAs, and the median of the EAs-positive samples was below 42.1 μg/kg. The positive samples also had rather broad EA concentration ranges. For instance, the ranges for total EAs and ergocristine were 21,969.2 and 12,414.6 μg/kg, respectively.

It was difficult to develop proper PLS models with such broad ranges and low concentrations. During the model construction stage, various attempts have been made to ameliorate the frequency distribution of EA content, such as removing samples with extremely high (e.g., >8000 ppb) or low (e.g., <10 μg/kg) concentrations of total or individual EAs.

### 2.2. Overview of Spectral Data

**Figure 1:**
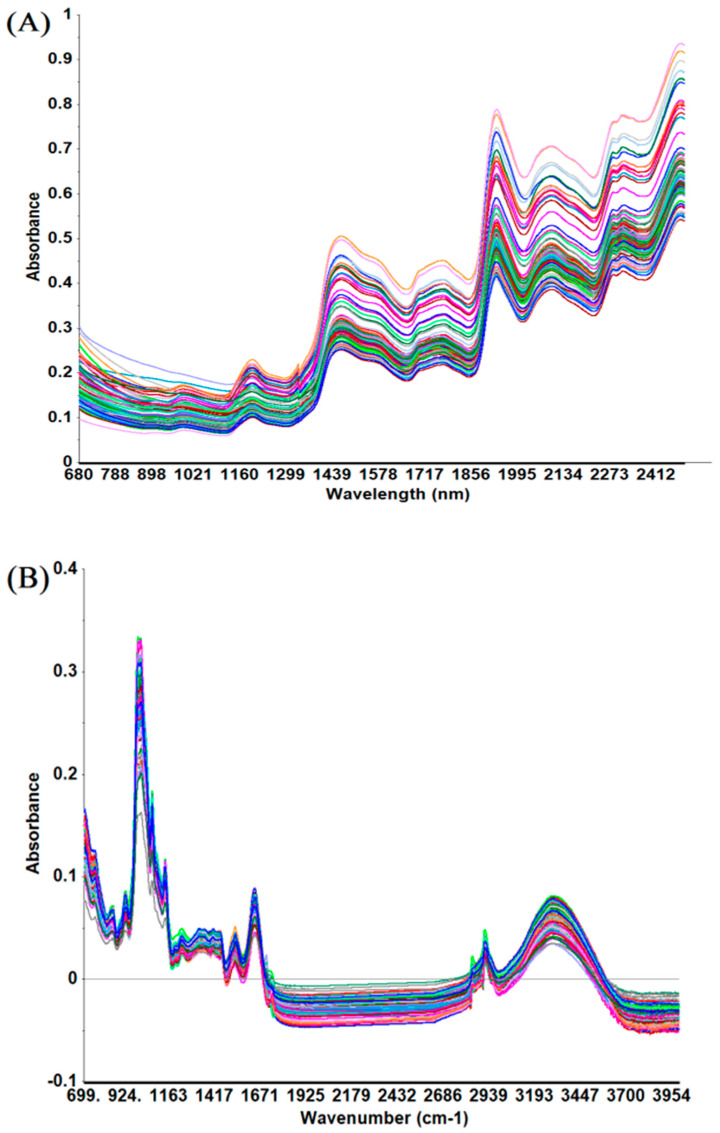
Infrared spectra of cool-season wheat grain samples grown under low heat unit (cold) climate conditions: (**A**) near-infrared range 680–2500 nm; (**B**) mid-infrared range 4000–700 cm^−1^.

The unprocessed IR spectra of wheat are shown in Figure 1. The peaks in the spectra were mainly due to absorption due to the presence of moisture, protein, carbohydrates, and lipids. NIR spectroscopy deals with molecular combination bands and overtones primarily of OH, CH, NH, and CO vibrations [13]. The broad and complex bands make it difficult to interpret the NIR spectrum visually and assign specific bands to specific chemical components. The MIR spectrum contains information related to fundamental molecular vibrations and could be divided into four major sections, including the region of X-H stretching (4000–2500 cm^−1^), triple bond (2500–2000 cm^−1^), double bond (2000–1500 cm^−1^), and the so-called “fingerprint” (1500–400 cm^−1^) [14]. In the NIR region, the characteristic absorption bands of proteins are located between 2148 and 2200 nm, which are related to combinations of C-O stretching, C-N stretching, N-H in-plane bending, the combination of C-H and C=O stretch, and the second N-H bend overtone [15]. Amide I (ca. 1700–1600 cm^−1^) and amide II (ca. 1480–1575 cm^−1^) bands were utilized for characterizing the primary structures and investigating the relative richness of protein molecules [10,16,17].

### 2.3. PLS Model Construction

The raw IR spectra usually contain undesirable noise and background variations, which can reduce the performance of multivariate models. Spectral variations unrelated to the chemical or physical properties of the samples can be removed by spectral pretreatment [15,18]. Both individual preprocessing and the integration of different preprocessing methods can be applied to reduce side information and multiple types of interference and variations [10,19].

Various pretreatments are available to researchers. For instance, non-uniform particle sizes in samples could result in light scattering effects; MSC and SNV are commonly used techniques to reduce such effects and adjust the baseline offsets. Resolution enhancement, random noise reduction, and subtle band shape highlights can be achieved by performing derivative algorithms. Detrending targets to adjust the curvilinearity and baseline shift of samples in powder form or densely packed samples [15,19,20].

In another research, several pretreatments including detrending, first derivative, SNV, SNV-Detrending, spectral-average, and baseline offset were used to preprocess the NIR spectra to develop PLS model for predicting endophyte alkaloids concentrations in dried perennial ryegrass [10,21].

The irrelevant information contained in full spectra may distort the models calibrated based on full wavelengths [19]. The redundancy and collinearity of the spectral data can be reduced by selecting important wavelengths. RCA is an effective technique for detecting important wavelengths [22]. Wavelengths with high absolute RC values are suggested as important wavelengths for the specific models.

**Figure 2:**
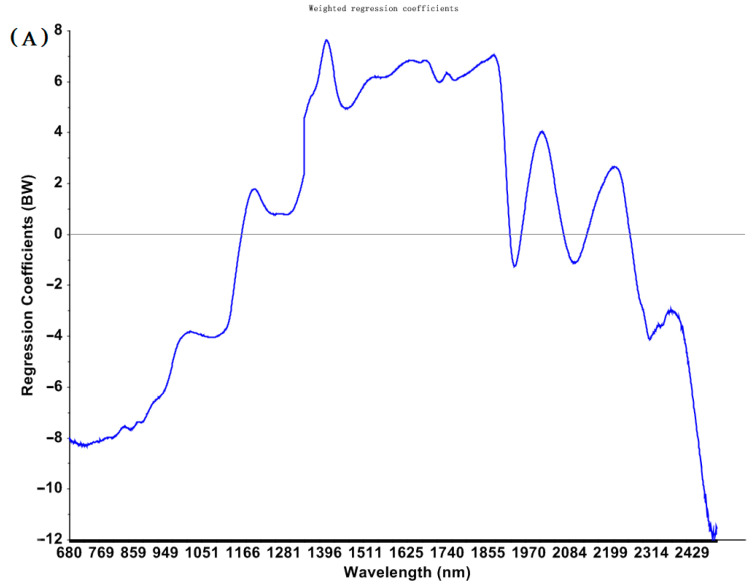

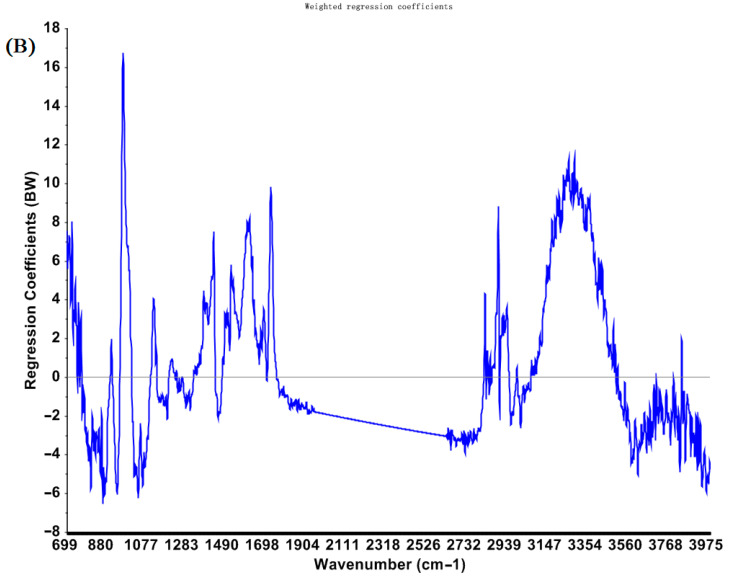
Regression coefficients for PLS models to predict total EAs content in cool−season wheat grain grown under low heat unit (cold) climate conditions constructed with SNV pretreated NIR (**A**) and MIR (**B**) spectra.

The RCA chart of PLS models established based on spectra preprocessed by SNV for predicting total EAs concentrations is shown in Figure 2. Several conditions, such as different pre-processing and different wavelengths (e.g., the fingerprint bands of the MIR region, the classical NIR bands of 1000–2500 nm, and a variety of selected sensitive wavelengths), were used for calibration to obtain acceptable PLS models.

### 2.4. Evaluation of PLS Models

Partial least squares regression is capable of reducing the data dimension and overcoming the multicollinearity problem and has been suggested as an alternative technique to ordinary least squares since the 1960s [10,23]. It is particularly powerful in developing IR models because it could effectively remove irrelevant spectral variations [24,25]. Many researchers have developed calibration models for predicting ergosterol, aflatoxin, fumonisin, ochratoxin A, and deoxynivalenol content in different cereals by the PLSR method or its variants [11].

**Figure 3:**
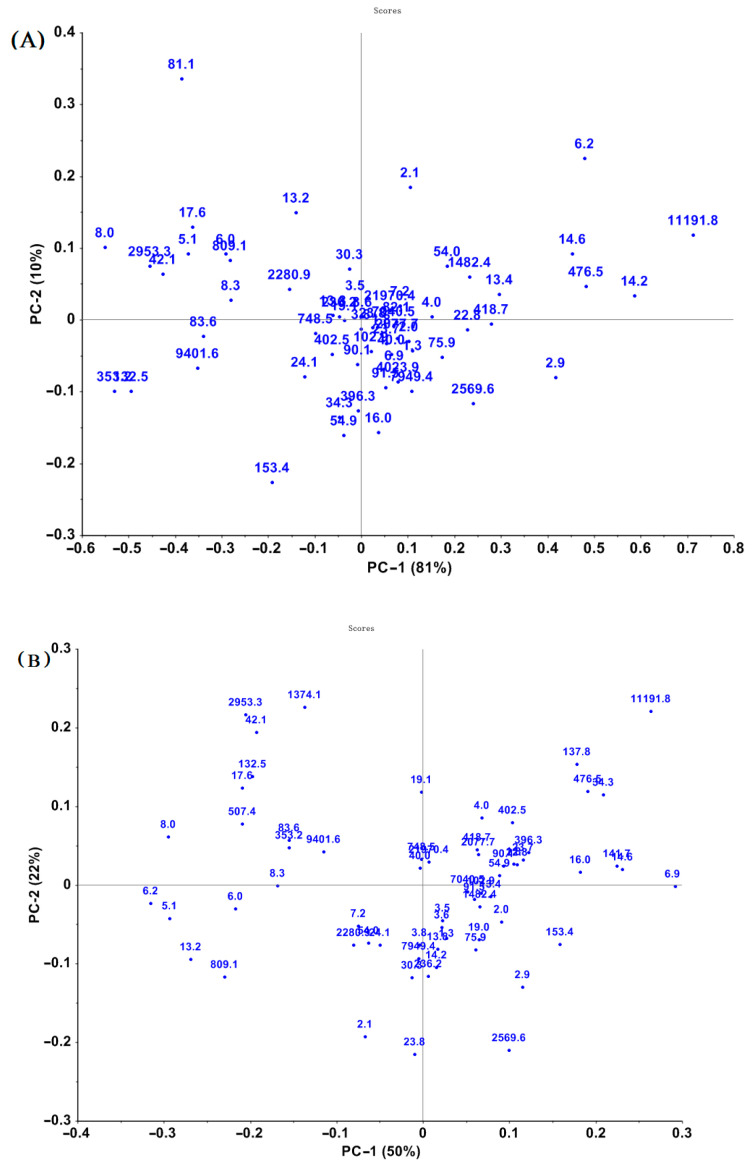
Principal component analysis (PCA) plots of spectral data of cool-season wheat grain samples grown under low heat unit (cold) climate conditions with different total EAs levels: NIR (**A**) and MIR (**B**) spectra preprocessed by MSC.

PCA was performed on both selected wavelength ranges and the full wavelength ranges to explore the sample spectral structures [10]. Nevertheless, the biplots of PCA analysis revealed that those wheat samples couldn’t be clustered clearly by EA concentration. The result of PCA analysis of the infrared spectra (pretreated by MSC) of samples containing different total EA concentrations is shown in Figure 3. The significant differences in the spectra of samples might result from differences in major chemical constituents among samples.

The performance of multivariate models can be evaluated with a number of statistical criteria. The coefficient of determination is a primary criterion that can indicate the goodness of fit [26]. Excellent models usually obtain a R^2^ greater than 0.91; the R^2^ of a good prediction ranges from 0.82 to 0.90; models with an R^2^ value between 0.66 and 0.81 could be used for approximate quantitative estimation; models with an R^2^ = 0.50–0.65 could only make discrimination analyses between samples with high and low concentrations [27].

The statistical parameters of NIR and MIR models constructed for predicting individual EAs and total EA concentrations are listed in Table 2 (1–7). Most PLS models developed in the present study obtained rather low R^2^_C_ values, and the R^2^_CV_ was unavailable (NA). Although the R^2^_C_ values of some models were higher (e.g., the model for ergocornine constructed with FD pretreated NIR spectra), their cross-validation statistics were very poor, and none of them had external prediction capability (i.e., the R^2^_P_ was unavailable). The results suggested that good calibration fit didn’t automatically produce desirable external predictive ability. This was inconsistent with the findings in a previous study, which reported that good fits to models during calibration do not infer the obtained model can make satisfying external predictions [28].

The statistical parameters obtained during the calibration and validation stages showed that the constructed models can’t be used for quantification or discrimination of the EA content in wheat. When the models were developed using a variety of selected wavelength ranges, no improvement was observed.

Roberts et al. (2005) [29] reported that the total EA concentrations in tall fescue can be determined by NIR spectroscopy. In their study, the EAs content analyzed by commercial ELISA test kits was calibrated with NIR spectra (1110–2490 nm) to create PLS models (R^2^ = 0.77–0.95). However, it should be noted that the actual values of total EAs were unavailable in their study, and the reference EA levels were replaced by the absorbance values since there was no standard for converting the relative content into actual EA concentrations. Furthermore, no independent prediction result was reported.

Lolitrem B, peramine, and ergovaline are common endophyte alkaloids found in perennial ryegrass plants. The NIR models for detecting and quantifying endophyte alkaloids in perennial ryegrass were constructed in a previous study [30]. The average levels for ergovaline, lolitrem B, and peramine were 0.71, 1.32, and 7.16 mg/kg, respectively. A modified PLS method was applied, and they obtained R^2^_CV_ values of 0.76, 0.41, and 0.94 for ergovaline, lolitrem B, and peramine, respectively. Based on their results, the concentration of peramine and ergovaline can be predicted by NIRS models, while the models for lolitrem B achieved undesired results.

In another study, the NIR hyperspectral imaging system was used to develop models for predicting ergot bodies in wheat [4]. They employed multivariate image analysis, PLS discriminant analysis, and support vector machine techniques to construct models. No false positives were observed with non-contaminated samples, and the LOD and LOQ were 145 mg/kg and 341 mg/kg, respectively. The greater ergot body concentration in those samples may facilitate the calibration process, although it should be noted that the EA type and levels in the ergot body can vary greatly. In another study, they reported that the discrimination models between ergot bodies and cereal kernels were constructed depending mainly on the differences in fat and starch levels of the grains [2]. Cereal kernels contain a high level of starch and a low level of fat, while ergot bodies are characterized by a high lipid content.

Based on the previous studies, the concentrations of EAs in the majority of samples might be too low for developing a proper NIR or MIR model. Moreover, the difference in IR absorbance bands between fungal-infected grains and healthy grains mainly reflects the changes in major chemical constituents such as carbohydrate, protein, etc. Many calibration studies indicated that the prediction of mycotoxin levels was not based on the toxin directly but rather relied on the spectral changes related to major chemical components [10,11].

The EA concentration in grains could be too low for direct determination by conventional NIR and MIR methods. Besides, the chemical profiles of ergot bodies may be different from those of normal grains, and the uneven distribution of ergot particles in grains could make it more difficult to obtain appropriate spectra for calibration and prediction.

## 3. Conclusions

The possibility of using NIR and ATR-FT/MIR techniques associated with various spectra preprocessing methods and wavelength ranges for the quantification of EAs in cool-season wheat was evaluated. During the calibration process, numerous spectral regions of both raw and preprocessed spectra were selected and calibrated, but the validation parameters of all PLS models were undesirable, and no model could be used to perform independent prediction. The EA content in most samples was rather low, which may be below the detectable limit of the employed IR spectroscopy. The frequency distribution of the EAs’ concentration was undesirable, which made the calibration more difficult. More research is needed in the future to explore the direct detection limit of the infrared spectroscopic methods for predicting EA concentrations in different grains.

## 4. Materials and Methods

### 4.1. Sample Preparation and LC-MS/MS Analysis

A total of 107 wheat samples grown in Western Canada were collected from May 2016 to August 2017. The determination of six major EAs was conducted at Prairie Diagnostic Services (PDS) by using the LC-MS/MS approach developed by Krska et al. (2008). Reagent EA standards were supplied by Romer Labs (Union, MO, USA). Primary-secondary amines were supplied by Agilent Technologies (Palo Alto, CA, USA) and employed as materials for dispersive solid-phase extraction. Acetonitrile and ammonium acetate were purchased from Fisher Scientific (Fair Lawn, NJ, USA). The standards for EAs were dissolved in acetonitrile and stored in a freezer (−80 °C).

A grinder with a 1.0 mm screen was used for grinding the samples. 5.0 g of the ground samples were weighed into an Erlenmeyer flask (125 mL). Twenty-five mL of 85:15 (*v*/*v*) acetonitrile/3.03 mM aqueous ammonium carbonate were added to the samples and stirred for 10 min. The supernatant was filtered into a clean beaker through Whatman No. 41 (ashless) filter paper. The filtrate (1 mL) was added to 50 mg of primary-secondary amine and agitated for 5 min to clean the matrix. The supernatant was used for EAs analysis.

The LC-MS/MS system was composed of an Agilent 1100 HPLC system and a Micromass Quattro Ultima^TM^ mass spectrometer (Waters, Milford, MA, USA). Multiple reaction monitoring was applied to identify the “parent” ions (first quadropole) and the “daughter” ions (second quadropole). The software used for data collection, processing, and curve construction was MassLynx 4.1 (Waters Corp., Milford, MA, USA). The standard curves were fitted to linear regression (*y* = *ax* + *b*), where x and y correspond to the alkaloids’ content and peak area, respectively. The recovery rates for ergometrine, ergometrine, ergocryptine, ergocornine, ergocristine, and ergosine were 51, 101, 81, 87, 75, and 82%, respectively. The limit of quantification was 1.25 μg/kg and the detection limit was 0.5 μg/kg. The concentration of total EAs is the sum of the six major EA concentrations. The detailed procedures and validation parameters of the methods could be found in another study [10,21].

### 4.2. NIR and MIR Spectra Collection

The Unity SpectraStar 2500XL-R NIR analyzer (Unity Scientific, Brookfield, CT, USA) was applied to obtain the NIR spectra in the reflectance mode from 680 nm to 2500 nm at an interval of 1 nm. The cool-season wheat samples were placed on the rotary sample-cup spinner. The NIR spectra (*.SPC format) were recorded with the built-in software (InfoStar, Unity Scientific, USA). The collection of MIR spectra (ca. 4000–700 cm^−1^) was carried out with the Jasco FT/IR-4200 spectrometer in attenuated total reflectance mode (JASCO Corp., Tokyo, Japan). To eliminate noise arising from water and carbon dioxide, the background spectra were recorded. The generated MIR spectra (in JWS format) were transformed to JCAMP-DX files by the JASCO Spectra Manager II software. For each sample, three replicate spectra were taken, and they were averaged prior to chemometric modeling. More detailed information regarding the spectra collection has been summarized in another study [9,10].

### 4.3. Chemometric Analysis

The Unscrambler^®^ X software (version 10.4, CAMO Software, Oslo, Norway) was applied to preprocess spectral data and perform the multivariate analysis.

Nine types of pretreatments were used to transform the raw spectra, including baseline offset, first and second order derivatives (FD and SD), the standard normal variate (SNV), multiplicative scattering correction (MSC), detrending, FD-SNV, SD-SNV, and SNV-detrending.

The spectral data structure and the potential outliers were explored by principal component analysis (PCA). Samples without outliers were classified into calibration and independent prediction sets in an approximate ratio of 3:1. Both the raw spectral data and the preprocessed spectral data were used to construct calibration models. The calibration models were developed based on calibration sets using the PLS algorithm. To investigate the important wavelength/wavenumber ranges, the regression coefficient analysis (RCA) was carried out using the Unscrambler software. Recalibrations were conducted using the selected sensitive wavelengths to optimize the predictive ability of the original models that were generated based on full wavelengths. Moreover, F-residuals and/or Hotelling’s T^2^ values were used for detecting the remaining outliers during regression stages. A leave-one-out cross-validation was performed to validate the established models. Furthermore, calibration models that obtained valid cross-validation parameters were applied to the individual prediction subsets to evaluate their potential for external prediction. More information regarding the modeling process is also available in another study [9,10].

To evaluate the PLS models, calibration statistics were calculated, including the coefficients of determination in calibration (R^2^_C_) and cross-validation (R^2^_CV_). The minimum values of root mean square error of calibration (RMSEC) and cross-validation (RMSECV) were used to select the best PLSR model [31]. The prediction determination coefficient (R^2^_P_), calibration root mean square error (RMSEP), and prediction standard error (SEP) were summarized for evaluating the prediction performance of the calibration models [10].

## Figures and Tables

**Table 1:** Statistical summary of reference ergot alkaloids content of wheat samples.

Parameter	Ergocornine	Ergocristine	Ergocryptine	Ergometrine	Ergosine	Ergotamine	Total EAs
N ^1^	37	64	53	45	36	44	75
Mean, %	97.48	743.31	142.07	150.59	56.88	337.48	1099.32
Max, %	1602.53	12,416.19	1954.23	1952.25	671.37	4462.75	21,970.40
Min, %	1.30	1.62	1.29	1.28	1.30	1.30	1.25
Median, %	5.63	39.26	11.30	11.20	7.07	33.21	42.10
Range, %	1601.23	12,414.57	1952.94	1950.97	670.07	4461.45	21,969.15
Standard deviation, %	296.89	1981.60	371.06	378.59	138.61	797.90	3221.62
Variance, %	88,142.30	3,927,756.00	137,686.30	143,327.80	19,212.86	636,637.90	10,378,850
Skewness	4.37	4.20	3.80	3.44	3.75	3.80	4.65

^1^ N: Number of samples contaminated with a quantifiable level of ergot alkaloids.

**Table 2:** 1. Parameters of partial-least square regression model using NIR and MIR techniques for the determination of ergocornine content in wheat. 2 (Cont’d). Parameters of a partial-least square regression model using NIR and MIR techniques for the determination of ergocristine content in wheat ^1^. 3 (Cont’d). Parameters of a partial-least square regression model using NIR and MIR techniques for the determination of ergocryptine content in wheat ^1^. 4 (Cont’d). Parameters of a partial-least square regression model using NIR and MIR techniques for the determination of ergometrine content in wheat ^1^. 5 (Cont’d). Parameters of a partial-least square regression model using NIR and MIR techniques for the determination of ergosine content in wheat ^1^. 6 (Cont’d). Parameters of a partial-least square regression model using NIR and MIR techniques for the determination of ergotamine content in cool-season wheat grain grown under low heat unit (cold) climate conditions ^1^. 7 (Cont’d). Parameters of a partial-least square regression model using NIR and MIR techniques for the determination of total ergot alkaloids content in cool-season wheat grain grown under low heat unit (cold) climate conditions ^1^.

**1**														
						**Calibration**			**Cross-Validation**			**External Prediction**		
**Pretreatment**	**Technique**	**N_C_**	**N_P_**	**Wavelength Range**	**Factor**	**R^2^_C_**	**RMSEC**	**SEC**	**R^2^_CV_**	**RMSECV**	**SECV**	**R^2^_P_**	**RMSEP**	**SEP**
NON	NIR	24	12	1700–2500 nm	1	0.02	350.35	357.89	NA	368.48	376.40	−	−	−
	MIR	24	12	1800–700 cm^−1^	1	0.24	310.82	317.51	NA	407.52	415.97	−	−	−
Baseline offset	NIR	24	12	680–2500 nm	1	0.05	344.90	352.32	NA	370.36	378.32	−	−	−
	MIR	24	12	1800–700 cm^−1^	1	0.17	324.02	330.99	NA	391.14	399.35	−	−	−
Detrending	NIR	24	12	680–2500 nm	1	0.05	344.95	352.37	NA	371.01	378.99	−	−	−
	MIR	24	12	1800–700 cm^−1^	1	0.17	324.47	331.45	NA	398.61	407.06	−	−	−
MSC	NIR	24	12	900–2000 nm	1	0.07	341.07	348.41	NA	391.96	400.39	−	−	−
	MIR	24	12	4000–700 cm^−1^	1	0.12	334.22	341.41	NA	410.75	419.58	−	−	−
SNV	NIR	24	12	1700–2500 nm	1	0.07	342.31	349.68	NA	381.52	389.72	−	−	−
	MIR	24	12	4000–700 cm^−1^	1	0.12	334.14	341.33	NA	410.74	419.57	−	−	−
SNV-Detrending	NIR	24	12	1100–2500 nm	1	0.13	329.97	337.07	NA	399.26	407.80	−	−	−
	MIR	24	12	3200–2700 cm^−1^	1	0.11	334.56	341.76	NA	415.97	424.91	−	−	−
FD	NIR	24	12	1200–1900 nm	1	0.04	347.05	354.52	NA	369.31	377.25	−	−	−
	MIR	24	12	2750–2950 cm^−1^	1	0.10	336.55	343.79	NA	372.15	380.15	−	−	−
SD	NIR	24	12	680–2500 nm	4	0.92	100.16	102.31	0.12	345.48	352.30	NA	242.72	227.66
	MIR	24	12	4000–700 cm^−1^	1	0.22	314.34	321.10	NA	383.78	392.03	−	−	−
FD-SNV	NIR	24	12	680–2500 nm	7	0.91	106.34	108.63	0.26	305.70	310.74	NA	275.39	271.23
	MIR	24	12	4000–700 cm^−1^	1	0.29	299.40	305.84	NA	422.64	431.73	−	−	−
SD-SNV	NIR	24	12	680–2500 nm	3	0.74	181.33	185.23	0.14	341.38	348.60	NA	132.71	137.16
	MIR	24	12	1800–700 cm^−1^	1	0.54	241.60	246.80	NA	408.13	416.85	−	−	−
**2**														
						**Calibration**			**Cross-Validation**			**External Prediction**		
**Pretreatment**	**Technique**	**N_C_**	**N_P_**	**Wavelength Range**	**Factor**	**R^2^_C_**	**RMSEC**	**SEC**	**R^2^_CV_**	**RMSECV**	**SECV**	**R^2^_P_**	**RMSEP**	**SEP**
NON	NIR	24	12	1300–2500 nm	1	0.04	1641.10	1676.39	NA	1843.45	1883.06	−	−	−
	MIR	24	12	1800–700 cm^−1^	1	0.10	1607.24	1641.80	NA	1799.57	1838.15	−	−	−
Baseline offset	NIR	24	12	1500–2500 nm	1	0.04	1641.90	1677.21	NA	1844.92	1884.47	−	−	−
	MIR	24	12	4000–700 cm^−1^	1	0.08	1621.82	1656.70	0.02	1749.88	1787.52	NA	1619.79	1686.22
Detrending	NIR	24	12	680–2500 nm	1	0.04	1644.06	1679.42	NA	1844.41	1883.88	−	−	−
	MIR	24	12	1800–700 cm^−1^	1	0.11	1595.34	1629.65	0.05	1746.87	1784.41	NA	1652.37	1720.66
MSC	NIR	24	12	1100–2300 nm	1	0.05	1636.33	1671.52	NA	1773.05	1811.18	−	−	−
	MIR	24	12	1800–700 cm^−1^	1	0.14	1572.20	1606.01	NA	1824.91	1864.11	−	−	−
SNV	NIR	24	12	1300–2200 nm	1	0.05	1636.02	1671.20	NA	1775.62	1813.81	−	−	−
	MIR	24	12	1800–700 cm^−1^	1	0.14	1571.49	1605.29	NA	1825.58	1864.80	−	−	−
SNV-Detrending	NIR	24	12	1100–2500 nm	1	0.06	1627.35	1662.35	NA	1861.23	1901.21	−	−	−
	MIR	24	12	1800–700 cm^−1^	1	0.15	1561.65	1595.24	NA	1838.60	1878.07	−	−	−
FD	NIR	24	12	1250–2250 nm	1	0.05	1639.82	1675.09	NA	1880.07	1920.31	−	−	−
	MIR	24	12	4000–700 cm^−1^	1	0.14	1571.35	1605.15	0.01	1759.46	1797.30	−	−	−
SD	NIR	24	12	1900–2500 nm	5	0.99	173.13	176.85	0.14	1623.09	1657.99	NA	1868.90	1937.85
	MIR	24	12	4000–700 cm^−1^	1	0.27	1447.99	1479.13	NA	1912.70	1953.59	−	−	−
FD-SNV	NIR	24	12	680–2500 nm	1	0.09	1597.72	1632.09	NA	1857.61	1897.39	−	−	−
	MIR	24	12	4000–700 cm^−1^	1	0.18	1534.38	1567.38	NA	1853.50	1892.76	−	−	−
SD-SNV	NIR	24	12	1250–2500 nm	3	0.76	819.45	837.08	0.14	1610.93	1645.00	0.01	1547.16	1594.61
	MIR	24	12	1800–700 cm^−1^	1	0.43	1281.08	1308.63	NA	2126.75	2165.27	−	−	−
**3**														
						**Calibration**			**Cross-Validation**			**External Prediction**		
**Pretreatment**	**Technique**	**N_C_**	**N_P_**	**Wavelength Range**	**Factor**	**R^2^_C_**	**RMSEC**	**SEC**	**R^2^_CV_**	**RMSECV**	**SECV**	**R^2^_P_**	**RMSEP**	**SEP**
NON	NIR	31	15	1200–2500 nm	1	0.03	364.82	368.85	NA	375.53	379.68	−	−	−
	MIR	19	9	1800–700 cm^−1^	3	0.47	405.06	416.16	0.16	526.69	540.99	NA	691.37	545.08
Baseline offset	NIR	31	15	1500–2500 nm	1	0.07	347.99	353.74	0.02	373.01	379.18	−	−	−
	MIR	19	9	1800–700 cm^−1^	1	0.35	448.32	460.60	0.12	533.11	547.52	NA	715.46	513.14
Detrending	NIR	31	15	680–2500 nm	1	0.06	348.59	354.35	NA	372.53	378.69	−	−	−
	MIR	19	9	1800–700 cm^−1^	3	0.45	411.93	423.21	0.13	546.94	561.49	NA	575.98	505.01
MSC	NIR	31	15	1700–2500 nm	1	0.09	343.16	348.84	NA	384.04	390.39	−	−	−
	MIR	19	9	1800–700 cm^−1^	3	0.49	398.18	409.09	0.20	521.37	535.26	NA	411.61	385.58
SNV	NIR	31	15	1500–2400 nm	1	0.10	342.20	347.86	NA	389.46	395.89	−	−	−
	MIR	19	9	1800–700 cm^−1^	3	0.49	397.94	408.85	0.20	521.76	535.58	NA	408.38	382.90
SNV-Detrending	NIR	31	15	900–2400 nm	1	0.12	337.72	343.31	NA	389.16	395.57	−	−	−
	MIR	19	9	1800–700 cm^−1^	3	0.47	402.38	413.40	0.22	500.03	513.66	NA	398.99	355.25
FD	NIR	31	15	1300–2000 nm	1	0.05	350.73	356.53	0.01	370.34	376.46	−	−	−
	MIR	19	9	4000–700 cm^−1^	2	0.54	375.79	386.09	0.13	543.90	558.59	NA	491.24	295.8
SD	NIR	31	15	1200–2500 nm	1	0.03	353.81	359.66	NA	369.32	375.43	−	−	−
	MIR	19	9	4000–700 cm^−1^	1	0.39	434.47	446.37	0.13	531.07	545.61	NA	518.52	273.73
FD-SNV	NIR	31	15	680–2500 nm	1	0.09	344.09	349.78	NA	376.95	383.18	−	−	−
	MIR	19	9	4000–700 cm^−1^	2	0.59	355.49	365.23	0.21	515.21	528.67	NA	375.43	228.24
SD-SNV	NIR	31	15	680–2500 nm	1	0.03	354.04	359.89	NA	370.82	376.95	−	−	−
	MIR	19	9	1800–700 cm^−1^	1	0.59	356.40	366.16	0.17	512.05	525.75	NA	488.11	313.50
**4**														
						**Calibration**			**Cross-Validation**			**External Prediction**		
**Pretreatment**	**Technique**	**N_C_**	**N_P_**	**Wavelength Range**	**Factor**	**R^2^_C_**	**RMSEC**	**SEC**	**R^2^_CV_**	**RMSECV**	**SECV**	**R^2^_P_**	**RMSEP**	**SEP**
NON	NIR	26	12	1100–2500 nm	1	0.04	436.89	445.54	NA	469.46	478.72	−	−	−
	MIR	30	14	1800–700 cm^−1^	1	0.01	417.36	424.50	NA	459.18	467.02	−	−	−
Baseline offset	NIR	26	12	1400–2500 nm	1	0.06	432.42	440.98	NA	467.49	476.71	−	−	−
	MIR	30	14	1800–700 cm^−1^	1	0.01	418.89	426.05	NA	447.97	455.58	−	−	−
Detrending	NIR	26	12	1300–2000 nm	1	0.06	431.64	440.19	NA	465.80	474.99	−	−	−
	MIR	30	14	1800–700 cm^−1^	1	0.01	418.27	425.42	NA	452.58	460.30	−	−	−
MSC	NIR	26	12	680–2500 nm	1	0.05	434.55	443.16	NA	500.46	510.07	−	−	−
	MIR	30	14	1800–700 cm^−1^	1	0.03	414.18	421.26	NA	466.45	474.42	−	−	−
SNV	NIR	26	12	680–2500 nm	1	0.05	434.58	443.19	NA	500.79	510.40	−	−	−
	MIR	30	14	1800–700 cm^−1^	1	0.03	414.27	421.35	NA	466.12	474.09	−	−	−
SNV-Detrending	NIR	26	12	1700–2400 nm	1	0.03	437.83	446.50	NA	476.78	486.22	−	−	−
	MIR	30	14	4000–700 cm^−1^	1	0.04	412.21	419.26	NA	475.95	484.05	−	−	−
FD	NIR	26	12	1200–2000 nm	1	0.07	430.40	438.93	NA	467.05	476.27	−	−	−
	MIR	30	14	1800–700 cm^−1^	1	0.02	414.83	421.92	NA	464.67	472.49	−	−	−
SD	NIR	26	12	680–2500 nm	1	0.01	442.32	451.08	NA	465.11	474.32	−	−	−
	MIR	30	14	1800–700 cm^−1^	1	0.18	379.45	385.94	NA	501.11	509.60	−	−	−
FD-SNV	NIR	26	12	1200–2300 nm	1	0.02	440.11	448.83	NA	467.84	477.11	−	−	−
	MIR	30	14	4000–700 cm^−1^	1	0.12	392.88	399.60	NA	502.98	511.57	−	−	−
SD-SNV	NIR	26	12	680–2500 nm	2	0.62	275.62	281.08	0.16	415.20	423.08	NA	433.66	373.51
	MIR	30	14	4000–700 cm^−1^	1	0.34	341.98	347.82	NA	508.91	517.61	−	−	−
**5**														
						**Calibration**			**Cross-Validation**			**External Prediction**		
**Pretreatment**	**Technique**	**N_C_**	**N_P_**	**Wavelength Range**	**Factor**	**R^2^_C_**	**RMSEC**	**SEC**	**R^2^_CV_**	**RMSECV**	**SECV**	**R^2^_P_**	**RMSEP**	**SEP**
NON	NIR	22	10	680–2500 nm	1	0.06	163.99	167.85	NA	175.88	180.02	−	−	−
	MIR	24	10	4000–700 cm^−1^	1	0.06	157.93	161.32	NA	179.19	183.05	−	−	−
Baseline offset	NIR	22	10	680–1900 nm	1	0.08	162.35	166.17	0.02	175.07	179.18	−	−	−
	MIR	24	10	1800–700 cm^−1^	1	0.08	156.78	160.15	NA	175.78	179.56	−	−	−
Detrending	NIR	22	10	680–2500 nm	1	0.07	163.14	166.98	NA	175.01	179.13	−	−	−
	MIR	24	10	1800–700 cm^−1^	1	0.05	158.79	162.21	NA	180.67	184.55	−	−	−
MSC	NIR	22	10	1400–2500 nm	1	0.06	164.21	168.07	NA	180.45	184.69	−	−	−
	MIR	24	10	1800–700 cm^−1^	1	0.04	160.23	163.68	NA	181.76	185.65	−	−	−
SNV	NIR	22	10	1800–2500 nm	1	0.06	163.72	167.58	NA	178.17	182.36	−	−	−
	MIR	24	10	4000–700 cm^−1^	1	0.04	160.23	163.68	NA	183.52	187.46	−	−	−
SNV-Detrending	NIR	22	10	1100–2500 nm	1	0.09	161.30	165.10	NA	179.45	183.67	−	−	−
	MIR	24	10	4000–700 cm^−1^	1	0.04	159.64	163.07	NA	184.87	188.83	−	−	−
FD	NIR	22	10	1250–2050 nm	1	0.07	163.42	167.26	NA	175.38	179.51	−	−	−
	MIR	24	10	1800–700 cm^−1^	1	0.05	158.89	162.31	NA	184.74	188.67	−	−	−
SD	NIR	22	10	1300–2500 nm	1	0.06	164.25	168.12	NA	175.34	179.46	−	−	−
	MIR	24	10	1800–700 cm^−1^	1	0.21	145.42	148.55	NA	183.62	187.16	−	−	−
FD-SNV	NIR	22	10	1200–2500 nm	1	0.06	163.68	167.54	NA	176.69	180.83	−	−	−
	MIR	24	10	4000–700 cm^−1^	1	0.17	148.01	151.19	NA	201.63	205.69	−	−	−
SD-SNV	NIR	22	10	680–2500 nm	3	0.79	77.66	79.49	0.22	151.28	154.78	NA	83.43	87.43
	MIR	24	10	4000–700 cm^−1^	1	0.43	123.63	126.29	NA	194.15	198.31	−	−	−
**6**														
						**Calibration**			**Cross-Validation**			**External Prediction**		
**Pretreatment**	**Technique**	**N_C_**	**N_P_**	**Wavelength Range**	**Factor**	**R^2^_C_**	**RMSEC**	**SEC**	**R^2^_CV_**	**RMSECV**	**SECV**	**R^2^_P_**	**RMSEP**	**SEP**
NON	NIR	28	13	680–2500 nm	1	0.02	915.72	932.52	NA	968.47	986.24	−	−	−
	MIR	19	9	1800–700 cm^−1^	1	0.09	991.25	1018.41	NA	1065.90	1095.09	−	−	−
Baseline offset	NIR	28	13	1400–2500 nm	1	0.03	913.28	930.03	NA	965.59	983.31	−	−	−
	MIR	19	9	4000–700 cm^−1^	1	0.07	1003.22	1030.71	NA	1079.52	1109.10	−	−	−
Detrending	NIR	28	13	1200–2200 nm	1	0.02	914.50	931.28	NA	964.52	982.22	−	−	−
	MIR	19	9	1800–700 cm^−1^	1	0.08	997.38	1024.71	0.05	1069.46	1098.76	−	−	−
MSC	NIR	28	13	1800–2400 nm	1	0.02	916.84	933.67	NA	970.83	988.63	−	−	−
	MIR	19	9	4000–700 cm^−1^	1	0.10	990.34	1017.48	NA	1086.01	1115.71	−	−	−
SNV	NIR	28	13	1200–2500 nm	1	0.02	917.60	934.43	NA	978.06	996.00	−	−	−
	MIR	19	9	4000–700 cm^−1^	1	0.10	989.93	1017.05	0.02	1085.99	1115.69	−	−	−
SNV-Detrending	NIR	28	13	680–2500 nm	1	0.02	914.15	930.92	NA	964.86	982.57	−	−	−
	MIR	19	9	4000–700 cm^−1^	1	0.10	985.34	1012.34	0.02	1088.02	1117.68	−	−	−
FD	NIR	28	13	680–2500 nm	1	0.03	913.61	930.18	NA	964.4	982.09	−	−	−
	MIR	19	9	1800–700 cm^−1^	1	0.09	991.35	1018.52	NA	1075.01	1104.45	−	−	−
SD	NIR	28	13	680–2500 nm	1	0.02	914.46	931.24	NA	967.61	985.36	−	−	−
	MIR	19	9	1800–700 cm^−1^	1	0.37	829.33	852.05	NA	1122.04	1152.23	−	−	−
FD-SNV	NIR	28	13	1200–2400 nm	1	0.02	914.74	931.52	NA	970.03	987.77	−	−	−
	MIR	19	9	4000–700 cm^−1^	1	0.15	961.67	987.67	NA	1134.22	1164.78	−	−	−
SD-SNV	NIR	28	13	1200–2500 nm	1	0.02	914.79	931.58	NA	967.85	985.60	−	−	−
	MIR	19	9	1800–700 cm^−1^	1	0.41	796.51	818.34	NA	1180.58	1210.74	−	−	−
**7**														
						**Calibration**			**Cross-Validation**			**External Prediction**		
**Pretreatment**	**Technique**	**N_C_**	**N_P_**	**Wavelength Range**	**Factor**	**R^2^_C_**	**RMSEC**	**SEC**	**R^2^_CV_**	**RMSECV**	**SECV**	**R^2^_P_**	**RMSEP**	**SEP**
NON	NIR	32	15	1500–2500 nm	1	0.01	2178.57	2213.43	NA	2377.60	2415.63	−	−	−
	MIR	32	16	1800–700 cm^−1^	1	0.09	1422.66	1445.42	NA	1731.73	1758.91	−	−	−
Baseline offset	NIR	32	15	1400–2500 nm	1	0.02	2169.78	2204.50	NA	2359.29	2397.03	−	−	−
	MIR	32	16	1800–700 cm^−1^	1	0.01	1484.59	1508.35	NA	1563.29	1588.30	−	−	−
Detrending	NIR	32	15	680–2500 nm	1	0.02	2174.16	2208.95	NA	2360.68	2398.44	−	−	−
	MIR	32	16	1800–700 cm^−1^	1	0.02	1476.51	1500.13	NA	1653.27	1679.72	−	−	−
MSC	NIR	32	15	1000–2400 nm	1	0.05	2142.26	2176.53	NA	2276.78	2313.21	−	−	−
	MIR	32	16	1800–700 cm^−1^	1	0.04	1466.07	1489.52	NA	1798.71	1827.05	−	−	−
SNV	NIR	32	15	1000–2500 nm	1	0.05	2142.06	2176.34	NA	2271.13	2307.42	−	−	−
	MIR	32	16	1800–700 cm^−1^	1	0.04	1463.25	1486.66	NA	1797.76	1825.54	−	−	−
SNV-Detrending	NIR	32	15	1200–2400 nm	1	0.07	2114.08	2147.91	NA	2398.58	2436.79	−	−	−
	MIR	32	16	1800–700 cm^−1^	1	0.06	1449.47	1472.66	NA	1871.50	1901.40	−	−	−
FD	NIR	32	15	1000–2300 nm	1	0.02	2174.77	2209.56	NA	2380.40	2418.48	−	−	−
	MIR	32	16	4000–700 cm^−1^	1	0.09	1423.08	1445.85	NA	1795.95	1823.84	−	−	−
SD	NIR	32	15	680–2500 nm	1	0.01	2188.35	2223.37	NA	2373.48	2411.46	−	−	−
	MIR	32	16	1800–700 cm^−1^	1	0.39	1163.46	1182.07	NA	1978.47	2006.52	−	−	−
FD-SNV	NIR	32	15	1000–2000 nm	1	0.06	2126.02	2160.04	NA	2367.78	2405.55	−	−	−
	MIR	32	16	1800–700 cm^−1^	1	0.25	1298.58	1319.36	NA	1932.25	1961.22	−	−	−
SD-SNV	NIR	32	15	1200–2500 nm	5	0.96	427.97	434.82	0.14	2099.56	2131.14	0.22	1585.45	1582.25
	MIR	32	16	1800–700 cm^−1^	1	0.28	1268.08	1288.37	NA	1797.17	1825.61	−	−	−

^1^ Abbreviation: N_C_, sample count of calibration set; N_p_, sample count of prediction set; R^2^_C_, coefficient of determination for calibration; RMSEC, root mean square error of calibration (%); SEC, standard error of calibration (%); R^2^_CV_, coefficient of determination for cross-validation (%); RMSECV, root mean square error of cross-validation (%); SECV, standard error of cross-validation (%); R^2^_P_, coefficient of determination for prediction (%); RMSEP, root mean square error of prediction (%); SEP, standard error of prediction (%); MSC, multiplicative scattering correction; SNV, standard normal variate; SNV-Detrending, SNV + detrending; FD-SNV, first derivative + SNV; SD-SNV, second derivative + SNV.

## Data Availability

The data presented in this study are available in this article.

## Abbreviations

General:
ATR-FT/MIR	attenuated total reflectance-Fourier transform mid-infrared spectroscopy
CP	crude protein
EAs	ergot alkaloids
IR	infrared
MIR	mid-infrared
NIR	near-infrared
PCA	principal component analysis
PLS	partial least square
RC	Regression coefficient
RCA	Regression coefficient analysis
Spectral Pretreatment Technique:
FD	first derivative
SNV	standard normal variate
FD-SNV	first derivative + SNV
MSC	multiplicative scattering correction
SNV-Detrending	SNV + detrending
SD-SNV	second derivative + SNV
SNV-SD	SNV + first derivative
Evaluate the models:
R^2^C	determination for calibration
R^2^CV	coefficient of determination for cross-validation
R^2^P	coefficient of determination for prediction
SEC	standard error of calibration
SECV	standard error of cross-validation
SEP	standard error of prediction
RMSEC	root mean square error of calibration
RMSECV	root mean square error of cross-validation
RMSEP	root mean square error of prediction
